# Arterial pressure variations as parameters of brain perfusion in response to central blood volume depletion and repletion

**DOI:** 10.3389/fphys.2014.00157

**Published:** 2014-04-23

**Authors:** Anne-Sophie G. T. Bronzwaer, Wim J. Stok, Berend E. Westerhof, Johannes J. van Lieshout

**Affiliations:** ^1^Department of Internal Medicine, Academic Medical Center, University of AmsterdamAmsterdam, Netherlands; ^2^Laboratory for Clinical Cardiovascular Physiology, Center for Heart Failure Research, Academic Medical CenterAmsterdam, Netherlands; ^3^Anatomy, Embryology and Physiology, Academic Medical Center, University of AmsterdamAmsterdam, Netherlands; ^4^Edwards Lifesciences BMEYEAmsterdam, Netherlands; ^5^MRC/Arthritis Research UK Centre for Musculoskeletal Ageing Research, Queen's Medical Centre, School of Life Sciences, University of Nottingham Medical SchoolNottingham, UK

**Keywords:** arterial pulse pressure, arterial systolic pressure, cerebrovascular circulation, fluid therapies, body fluids, head-up tilt, spontaneous breathing

## Abstract

**Rationale:** A critical reduction in central blood volume (CBV) is often characterized by hemodynamic instability. Restoration of a volume deficit may be established by goal-directed fluid therapy guided by respiration-related variation in systolic- and pulse pressure (SPV and PPV). Stroke volume index (SVI) serves as a surrogate end-point of a fluid challenge but tissue perfusion itself has not been addressed.

**Objective:** To delineate the relationship between arterial pressure variations, SVI and regional brain perfusion during CBV depletion and repletion in spontaneously breathing volunteers.

**Methods:** This study quantified in 14 healthy subjects (11 male) the effects of CBV depletion [by 30 and 70 degrees passive head-up tilt (HUT)] and a fluid challenge (by tilt back) on CBV (thoracic admittance), mean middle cerebral artery (MCA) blood flow velocity (V_mean_), SVI, cardiac index (CI), PPV, and SPV.

**Results:** PPV (103 ± 89%, *p* < 0.05) and SPV (136 ± 117%, *p* < 0.05) increased with progression of central hypovolemia manifested by a reduction in thoracic admittance (11 ± 5%, *p* < 0.001), SVI (28 ± 6%, *p* < 0.001), CI (6 ± 8%, *p* < 0.001), and MCAV_mean_ (17 ± 7%, *p* < 0.05) but not in arterial pressure. The reduction in MCAV_mean_ correlated to the fall in SVI (*R*^2^ = 0.52, *p* < 0.0001) and inversely to PPV and SPV [*R*^2^ = 0.46 (*p* < 0.0001) and *R*^2^ = 0.45 (*p* < 0.0001), respectively]. PPV and SPV predicted a ≥15% reduction in MCAV_mean_ and SVI with comparable sensitivity (67/67% vs. 63/68%, respectively) and specificity (89/94 vs. 89/94%, respectively). A rapid fluid challenge by tilt-back restored all parameters to baseline values within 1 min.

**Conclusion:** In spontaneously breathing subjects, a reduction in MCAV_mean_ was related to an increase in PPV and SPV during graded CBV depletion and repletion. Specifically, PPV and SPV predicted changes in both SVI and MCAV_mean_ with comparable sensitivity and specificity, however the predictive value is limited in spontaneously breathing subjects.

## Introduction

Severe hypovolemia is associated with a critical reduction in central blood volume (CBV) quite often related to hemorrhage or dehydration. This results in hemodynamic instability with a reduction in cardiac output (CO) and tissue oxygen delivery.

Volume depletion is usually of acute onset, and neurocardiovascular control mechanisms including reflexes from high and low pressure area receptors initiate the body's defending response (Guyton et al., 1980). In contrast, in chronic hypovolemia, the capillary fluid shift transfers fluid to the intravascular space (Guyton, 1980), whereas the humoro-cardiovascular and long-term renal blood volume control systems with a longer time constant come into operation (Shepherd and Vanhoutte, 1979; DiBona and Wilcox, 1992).

Restoration of adequate tissue perfusion and oxygenation is of major importance in hemodynamically unstable patients. In anesthesia and intensive care medicine, establishing this therapeutic goal typically involves intravascular fluid administration as the cornerstone of treatment for central hypovolemia.

However, diagnosing a volume deficit is not straightforward. In present clinical practice, volume treatment is commonly adjusted by recordings of the heart rate (HR) and arterial blood pressure (BP). The experience is, however, that fluid infusion by focusing on these hemodynamic variables allows for wide variation in administered volume because neither BP nor HR accurately reflects changes in CBV (Secher and Van Lieshout, 2005; Bundgaard-Nielsen et al., 2007; Maizel et al., 2007; Secher and Van Lieshout, 2010). Observations in hypotensive patients during hemorrhage indicate that reversible hypotensive hypovolemic shock is in fact characterized by a decrease in HR reflecting an increase in vagal tone (Sander-Jensen et al., 1986). Thus the sensitivity of HR as an early indicator is low and highly non-specific. This is further supported by data obtained in a human model of acute hypovolemic shock by either lower body negative pressure (Cooke et al., 2004; Rickards et al., 2014) or passive head-up tilt (Matzen et al., 1991; Ten Harkel et al., 1992; Westerhof et al., 2006).

Also, clinical signs of hypovolemia including diminished skin turgor and high urine osmolarity do not accurately reflect reductions in CBV (McGee et al., 1999).

An increase in stroke volume (SV) or CO in response to fluid therapy is considered favorable. A meta-analysis of 12 clinical studies showed that with current clinical practice, between 40 and 70% of critically ill patients are so-called responders (Michard and Teboul, 2002). The substantial number of patients not responding to fluid therapy calls for physiological monitors capable of predicting fluid responsiveness.

Respiration-related variations in left ventricular preload which are transferred to variations in arterial pressure [e.g., systolic pressure variation (SPV) and pulse pressure variation (PPV)] are being introduced in clinical medicine as potentially useful tools to guide volume administration (Michard et al., 2000; Michard and Teboul, 2002; Bendjelid and Romand, 2003; Preisman et al., 2005).

In the majority of studies aiming for candidate indices predictive for fluid responsiveness, however, the investigated end-point of a fluid challenge has been a change in SV (index; SVI) or CO / cardiac index (CI) (Marik et al., 2009).

Although SV and CO serve as surrogate end-points of a fluid challenge, brain perfusion as the actual therapeutic endpoint is as yet not being addressed by present research. The large metabolic needs of the brain reflected by respectively 20 and 25% of oxygen and glucose consumption by neuronal activity renders it extremely sensitive to sufficient and uninterrupted blood supply. In this study, the hypothesis is tested that arterial pressure variations during progressive central hypovolemia relate to changes in brain perfusion. We therefore set out to gauge the relationship between arterial pressure variations, SVI and cerebral blood flow velocity during CBV depletion and repletion in spontaneously breathing subjects.

## Methods

### Subjects

Fourteen healthy volunteers (11 males) with a median (range) age of 25 (23–37) year, height 180 (173–204) cm and weight 72 (62–86) kg, without taking any medication and/or history of regular fainting or cardiac arrhythmia participated in this study. Phase of menstrual cycle in female subjects was not accounted for. This study was approved by the institutional Medical Ethics Committee and took place in the Laboratory for Clinical Cardiovascular Physiology in the Academic Medical Center in Amsterdam. The subjects abstained from heavy physical exercise and caffeinated beverages 4 h prior to the experiment. Diurnal variations in body fluid contents were accounted for by strictly adhering the experiment to the same hour of the day. All procedures and risks associated with the study were explained to the subjects and written informed consents were obtained.

### Experimental protocol

Measurements were performed between 11 am and 3 pm in a quiet room with the subjects on a custom built computer controlled tilt table that minimizes muscle tensing and limits vestibular stimulation during tilting (Gisolf et al., 2004a). Resting supine measurements represented normovolemic conditions. Next, subjects were head-up tilted (HUT) to respectively 30 and 70 degrees causing progressive central hypovolemia of acute onset and subsequently tilted back to the supine position mimicking a rapid volume repletion. Following each angle change of the tilt table, that body position was maintained for five minutes to obtain a stable hemodynamic situation. The last 60 s of these adjustment periods were used for analysis. Following tilt-back to the supine position, the subjects rested again. Subsequently they were tilted in a sinusoidal fashion (tilting frequency varying from 0.042 to 0.2 Hz or 5 to 12/min) enhancing BP variation for evaluation of cerebrovascular autoregulatory efficacy. The breathing was paced at 13 breaths/min by auditory support. This breathing frequency was continued throughout the sinusoidal tilts, to separate the influence of gravity and the influence of respiration on the measured cardiovascular signals.

### Measurements

**Figure 1:**
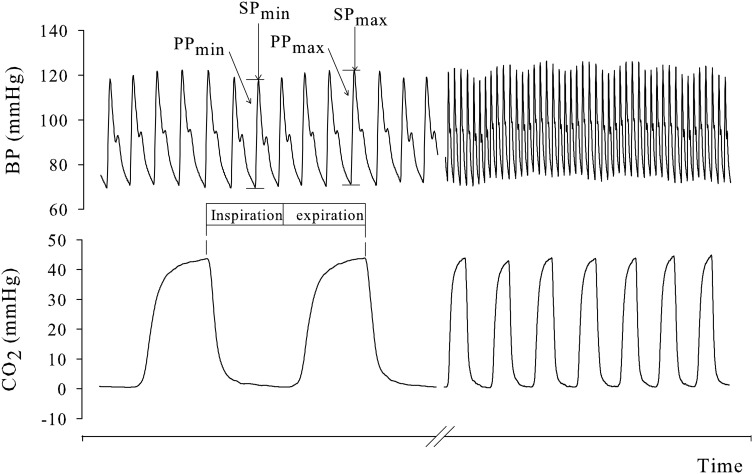
**Variation in pulse and systolic pressure (PP and SP, respectively), induced by respiration**. PPV and SPV were first calculated (Equation 1) per breath and then averaged over five consecutive breaths.

Continuous arterial BP was non-invasively measured (Nexfin, Edwards Lifesciences BMEYE, Amsterdam, the Netherlands) (Eeftinck Schattenkerk et al., 2009; Martina et al., 2012) using the volume clamp method (Truijen et al., 2012). A finger cuff fastened on the middle finger was held at heart level. A pulse contour method (Nexfin CO-trek, Edwards Lifesciences BMEYE, Amsterdam, the Netherlands)—adapted for age, gender, height and weight (Bogert et al., 2010)—provided left ventricular SV and CO by multiplying SV by instantaneous HR. SVI and CI were SV and CO divided by body surface area (Du Bois and Du Bois, 1916). SPV and PPV were calculated per breath from the BP signal according to the following formula:

(1) $$100\times \frac{{\text{A}}_{\max}-{\text{A}}_{\min}}{\left({\text{A}}_{\max}+{\text{A}}_{\min}\right)/2}$$

with A equal to, respectively, systolic arterial pressure (SAP) and pulse pressure [PP; equal to SAP minus diastolic arterial pressure (DAP)]. Arterial pressure variations were calculated per breath and then averaged over five consecutive breaths (see Figure 1).

Cerebral perfusion pressure (CPP) was determined by subtracting critical closing pressure (CCP) from BP at brain level. BP at brain level was estimated by subtracting hydrostatic difference between the finger cuff and the level of transcranial insonation by Transcranial Doppler (TCD) ultrasonography and CCP was estimated by first harmonic Fourier filtered normal arterial pressure and velocity wave heart beat data as described by Aaslid et al. (2003).

Regional brain perfusion of the anterior circulation was investigated by the assessment of mean middle cerebral artery (MCA) flow velocity (V_mean_) followed in the proximal segments by means of TCD (DWL Multidop X4, Sipplingen, Germany). The MCA was insonated through the temporal window just above the zygomatic arch at a depth of 40–60 mm with a pulsed 2 MHz probe. After the Doppler signal was optimized, the probe was attached to the skull in a fixed angle by means of a head-band.

The HUT induced translocation of CBV to the lower extremities was monitored by electrical impedance plethysmography (350 μA at 50 kHz; Nihon Kohden, AI-601G, Japan) measured at the level of the thorax (Krantz et al., 2000) and expressed as changes in thoracic admittance (Van Lieshout et al., 2005). Airway flow and pressure were measured by means of the Alveotest flowmeter (Jaeger, Würzburg, Germany) and end-tidal CO_2_ (PetCO_2_) was measured using a capnograph (Tonocap, Datex-Ohmeda, Madison, USA). Tidal volume (TV) was calculated by integration of the flow signal. All data were sampled at 200 Hz and collected using an Analog Devices RTI815 PC-card with custom made software. Signals were visually inspected for artifacts and analyzed offline (Matlab R2007b, Mathworks Inc. MA, USA).

Dynamic cerebral autoregulation (CA) was quantified as the counter-regulatory capacity to maintain MCAV_mean_ during oscillatory tilt induced changes in BP. Dynamic CA was assessed by means of cross-spectral density analysis of beat-to-beat data of MAP at brain level and MCAV_mean_ after spline interpolation and resampling at 4 Hz. Gain and phase were obtained for the frequencies equal to the oscillatory tilt frequencies (ranging from 0.042 to 0.2 Hz). The gain reflects the effective amplitude dampening of BP fluctuations and phase shift was defined positive where MCAV_mean_ leads MAP at brain level. Coherence examined the strength of the relationship between MAP and MCAV_mean_ (Immink et al., 2004). To account for inter-subject variability, the gain was normalized for MAP and MCAV_mean_ and expressed as the percentage change in cm/sec per percentage change in mmHg (Panerai et al., 1999).

### Statistical analysis

Results are presented as mean ± *SD*. The effect of HUT on measured parameters was assessed using a One Way Repeated Measures Analysis of Variance (ANOVA) test together with the Holm-Sidak method to perform pairwise multiple comparisons. When data were not normally distributed, they are presented as medians and range and non-parametric statistical tests were used.

Linear Mixed Model analysis was performed (IBM SPSS statistics 20, IBM corporation, USA) to examine the relation between brain perfusion (referred to as dependent variable) and hemodynamic and respiratory variables (fixed covariates). *R*^2^ was calculated according to the following formula:

(2) $$R^{2}=1-\frac{\sum_{i}{\left(y_{i}-f_{i}\right)}^{2}}{\sum_{i}{\left(y_{i}-\bar{y}\right)}^{2}}$$

where *y* and *f* refers to, respectively, the observed and predicted values (Edwards et al., 2008). A multivariate, stepwise regression model was constructed with MCAV_mean_ as the dependent variable and mean arterial pressure at heart level (MAP), CPP, SVI, HR, total peripheral resistance (TPR), PetCO_2_ and thoracic admittance as the independent variables (Kim et al., 2008). The model was developed by forward entry and removal of the independent variables according to their significant contribution (according to the *F*-test) in explaining the variance in the dependent variable. Baseline measurements for both the dependent as independent variables were normalized to zero and the effect of tilt was expressed as absolute change with respect to the baseline value.

The subjects were divided into two groups according to the percent decrease in SVI or MCAV_mean_ during progressive CBV depletion related to the resting supine value. A 15% increase in SVI in response to fluid infusion is considered clinically relevant according to previously published criteria (Michard et al., 2000; Heenen et al., 2006; Jellema et al., 2006; Soubrier et al., 2007). In this study, subjects with >15% decrease in SVI or MCAV_mean_ were classified as having a CBV deficit. Receiver operating characteristics (ROC) curves evaluated the predictive value of PPV and SPV on volume deficits by determination of sensitivity and specificity values and its corresponding optimal threshold value (Akobeng, 2007). Accuracy was assessed by the area under the curve (AUC) values presented as area ± *SD*. A *p*-value less than 0.05 was considered to indicate a statistically significant difference.

## Results

**Figure 2:**
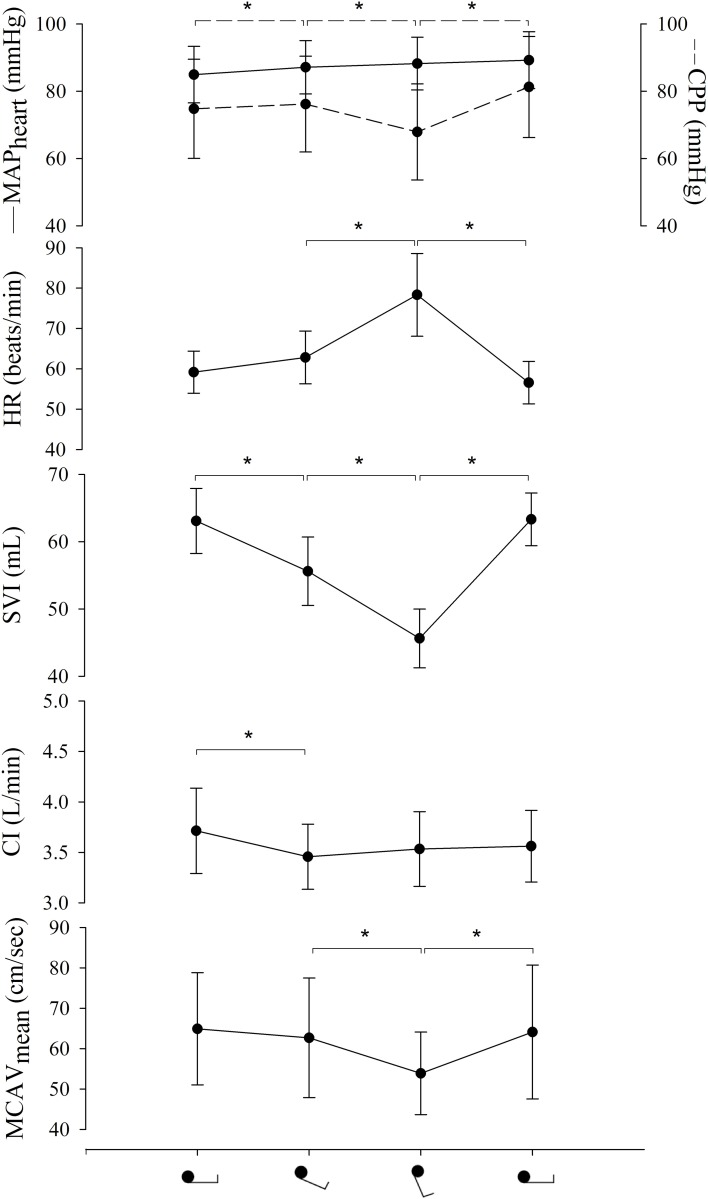
**Hemodynamic response to graded HUT and tilt back**. MAP, mean arterial pressure; CPP, cerebral perfusion pressure; HR, heart rate; SVI, stroke volume index; CI, cardiac index; MCAV_mean_, mean middle cerebral artery flow velocity. ^*^p < 0.001.

**Figure 3:**
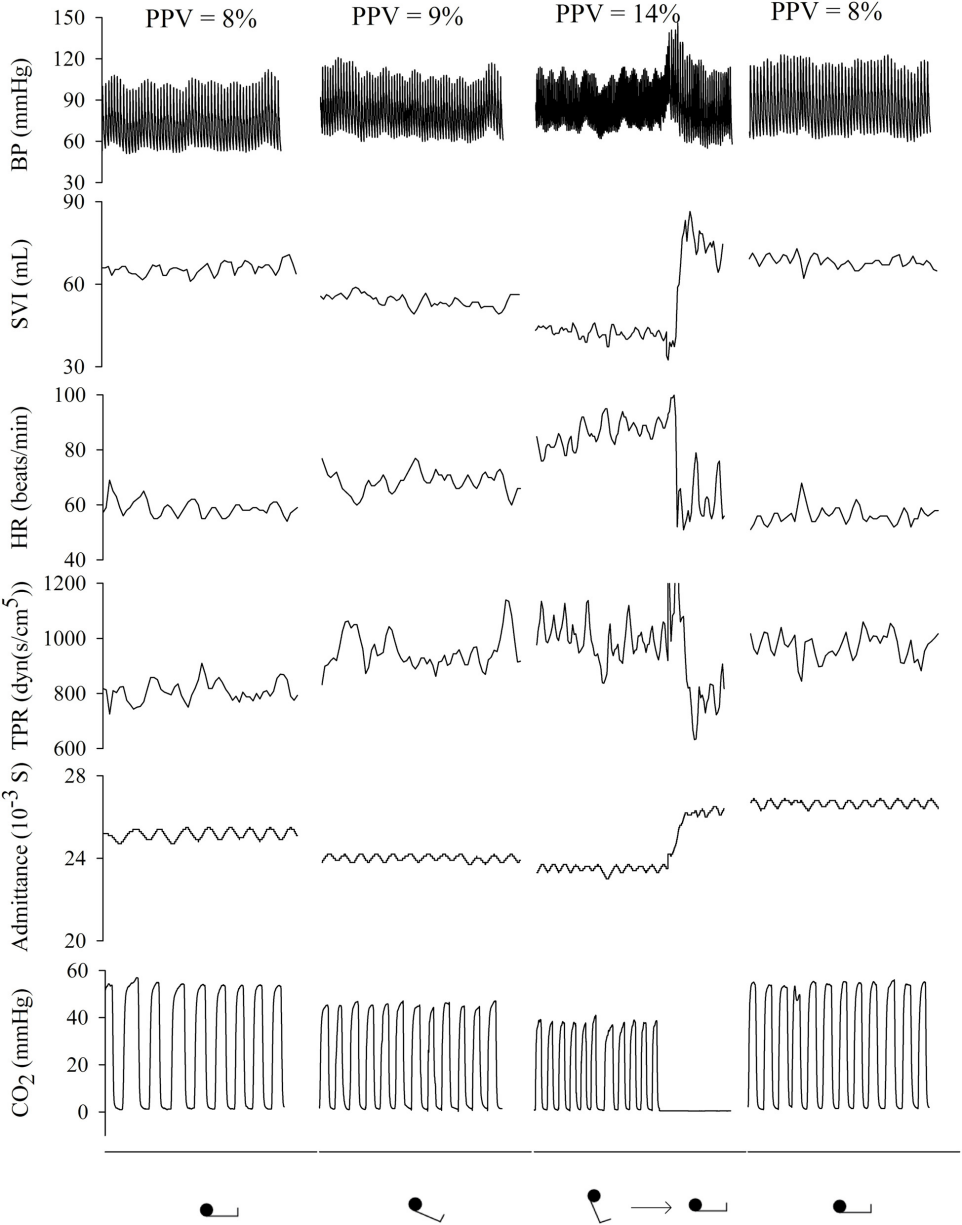
**Representative example (subject 11) of hemodynamic response to graded HUT and tilt back**. BP, blood pressure; SVI, stroke volume index; HR, heart rate; TPR, total peripheral resistance; PPV, pulse pressure variation.

All subjects completed the protocol. Figures 2, 3 and Table 1 summarize the hemodynamic and brain perfusion response to graded HUT and tilt back.

**Table 1:** **Effect of tilt on measured parameters**.

		**Supine**	**Volume depletion**		**Volume repletion**
		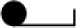	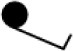	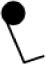	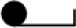
MAP	(mmHg)	85 ± 8	87 ± 8	88 ± 8	89 ± 8
SAP	(mmHg)	120 ± 12	119 ± 11	116 ± 10	122 ± 9
DAP	(mmHg)	66 ± 6	69 ± 5	73 ± 6^*^	69 ± 6
PP	(mmHg)	55 ± 7	50 ± 7^*^	44 ± 5^*^	53 ± 5^*^
CPP	(mmHg)	75 ± 15	76 ± 14	68 ± 14	81 ± 15^*^
HR	(beats/min)	59 ± 5	63 ± 7^*^	78 ± 10^*^	57 ± 5^*^
SVI	(mL/m^2^)	64 ± 4	56 ± 5^*^	46 ± 4^*^	63 ± 4^*^
CI	[(L/min)/m^2^]	3.7 ± 0.4	3.5 ± 0.3^*^	3.5 ± 0.4	3.6 ± 0.4
T. adm.	(10^−3^ S)	29 ± 3	27 ± 3^*^	26 ± 3^*^	29 ± 3^*^
TPR	dyn(s/cm^5^)	962 ± 165	1052 ± 145^*^	1053 ± 185	1052 ± 156^‡^
MCAV_mean_	(cm/s)	65 ± 14	63 ± 15	54 ± 10^*^	64 ± 17^*^
PPV	(%)	11 ± 4	12 ± 3	20 ± 5^*^	10 ± 3^*^
SPV	(%)	6 ± 3	6 ± 2	11 ± 3^*^	5 ± 2^*^
TV	(mL/Kg)	11 ± 3	11 ± 3	11 ± 3	10 ± 3
PetCO_2_	(mmHg)	39 ± 5	39 ± 3	35 ± 4^*^	38 ± 4^*^

Values are presented as mean ± SD.

*MAP, mean arterial pressure; SAP, systolic arterial pressure; DAP, diastolic arterial pressure; PP, pulse pressure; CPP, cerebral perfusion pressure; HR, heart rate; SVI, stroke volume index; CI, cardiac index; T.adm., thoracic admittance; TPR, total peripheral resistance; MCAV*_mean_*, mean middle cerebral artery flow velocity; PPV, pulse pressure variation; SPV, systolic pressure variation; TV, tidal volume; PetCO_2_, end-tidal CO_2_.*

* p < 0.05 with respect to the previous tilt position.

‡ p < 0.05 volume repletion vs. baseline supine position.

### Central blood volume depletion (HUT)

Mean arterial pressure and SAP remained constant while DAP increased (11 ± 12%, *p* < 0.001) with 70 degrees HUT resulting in a 19 ± 11% (*p* < 0.001) reduction in PP. Although a decrease in CPP was demonstrated during HUT, this was not significantly different from the supine position. SVI (28 ± 6%, *p* < 0.001), thoracic admittance (11 ± 5%, *p* < 0.001) and MCAV_mean_ (17 ± 7%, *p* < 0.05) declined while an increase was seen in HR (32 ± 14%, *p* < 0.001). CI declined (6 ± 8%, *p* < 0.001) and TPR increased (10 ± 11%, *p* < 0.001) only from 0 to 30 degrees without further change at 70 degrees HUT. PPV and SPV did not change from supine to 30 degrees HUT but substantially increased (103 ± 89% (*p* < 0.05) and 136 ± 117% (*p* < 0.05), respectively) with 70 degrees HUT. TV and breathing frequency did not change during HUT vs. the supine position while PetCO_2_ declined (8 ± 11%, *p* < 0.001) with 70 degrees HUT.

### Central blood volume repletion (tilt back)

Every changing parameter returned to baseline values following tilt back except TPR which remained elevated compared to the baseline measurement (*p* < 0.05, Table 1).

### Cerebral blood flow velocity and stroke volume

**Figure 4:**
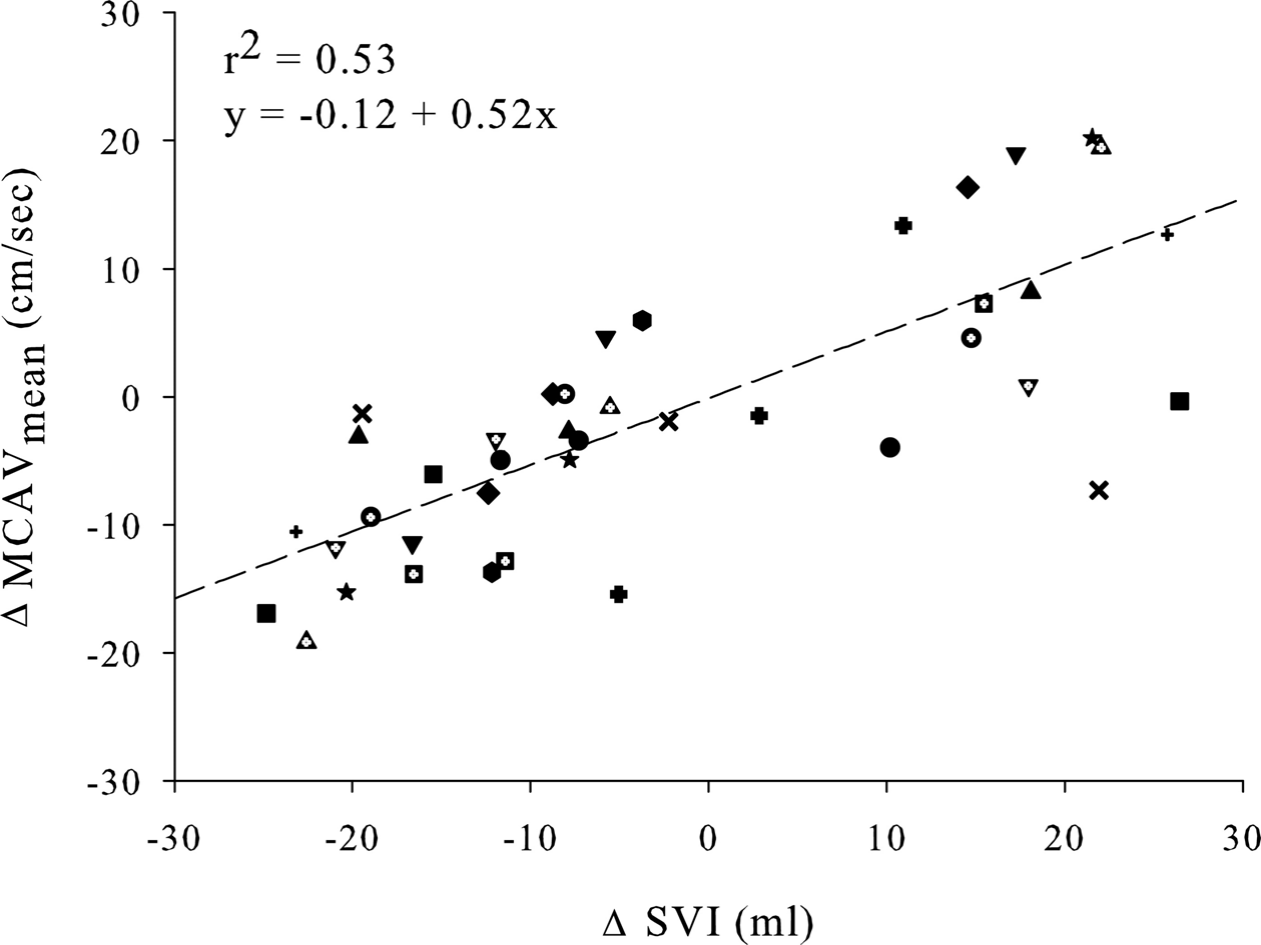
**Linear regression plot of absolute change in MCAV_mean_ and SVI**. All subjects contributed three points in each regression line (during 30 and 70 degrees + 0 degrees after tilt back). The regression function is equal to *y* = −0.12 + 0.52x with corresponding correlation coefficient of *r*^2^ = 0.53 (*p* < 0.0001). MCAV_mean_, mean middle cerebral artery flow velocity; SVI, stroke volume index.

**Figure 5:**
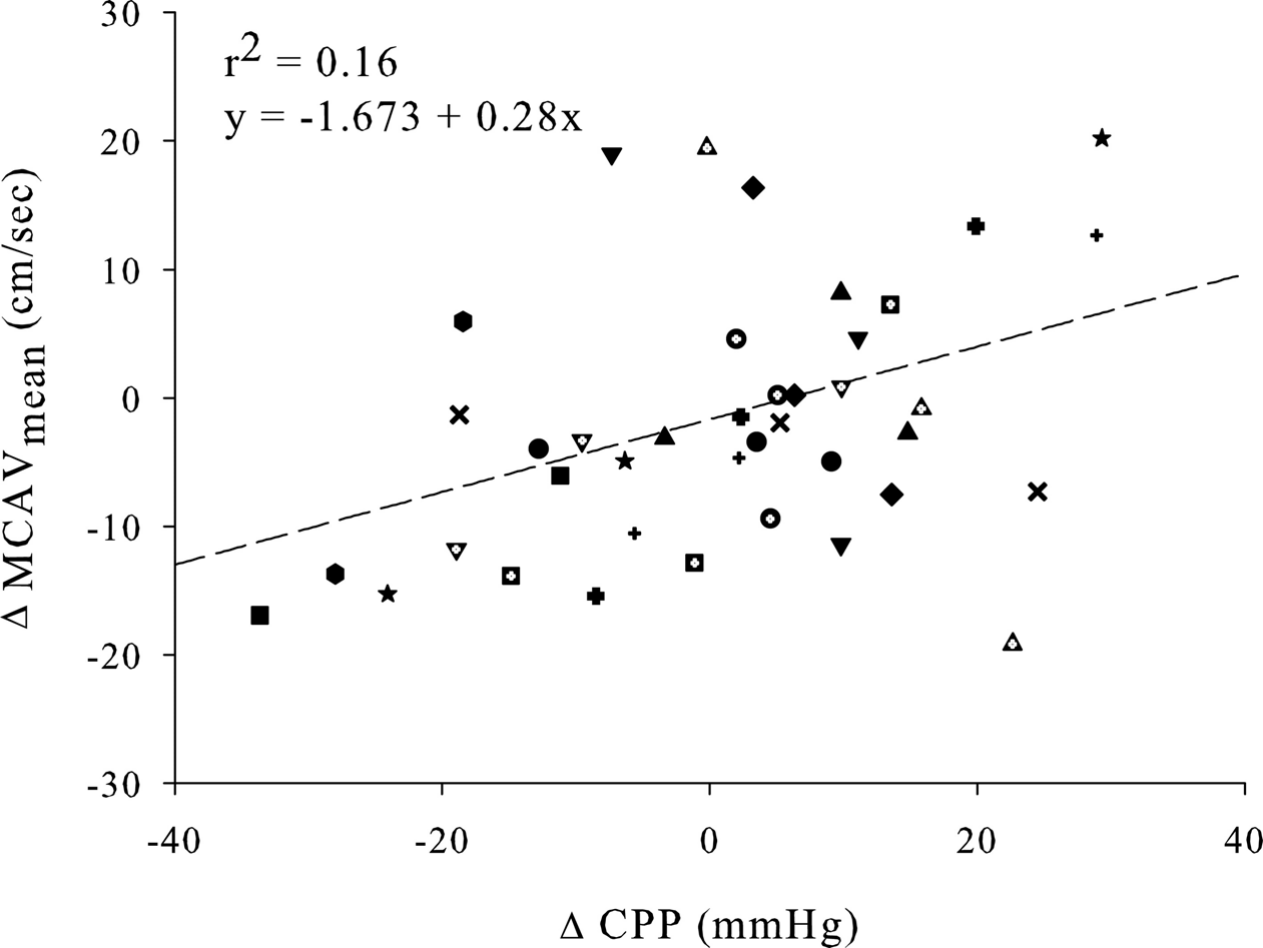
**Single linear regression plot of absolute change in MCAV_mean_ and CPP**. All subjects contributed three points in each regression line (during 30 and 70 degrees + 0 degrees after tilt back). The regression function is equal to *y* = −1.673 − 0.28x with corresponding correlation coefficient of *r*^2^ = 0.16 (*p* = 0.007). MCAV_mean_, mean middle cerebral artery flow velocity; CPP, cerebral perfusion pressure.

Forward stepwise regression analysis revealed that absolute change in MCAV_mean_ was mainly predicted by absolute change in SVI (*r*^2^ = 0.53, *p* < 0.001). Absolute change in PetCO_2_ was secondly added to this prediction model, resulting in a slightly stronger regression (*r*^2^ = 0.57, *p* < 0.001). Single linear plots of the dependent variable (MCAV_mean_) and its strongest prediction variable (SVI) are shown in Figure 4. There was only a weak relation between CPP and MCAV_mean_ (*r*^2^ = 0.16, *p* = 0.007; Figure 5). The median (range) of individual correlation coefficients for the relation between SVI and MCAV_mean_ and for CPP and MCAV_mean_ was: *r*^2^ = 0.902 (0.059–0.999) and *r*^2^ = 0.345 (−0.672–0.998) respectively.

### Arterial pressure variations

**Figure 6:**
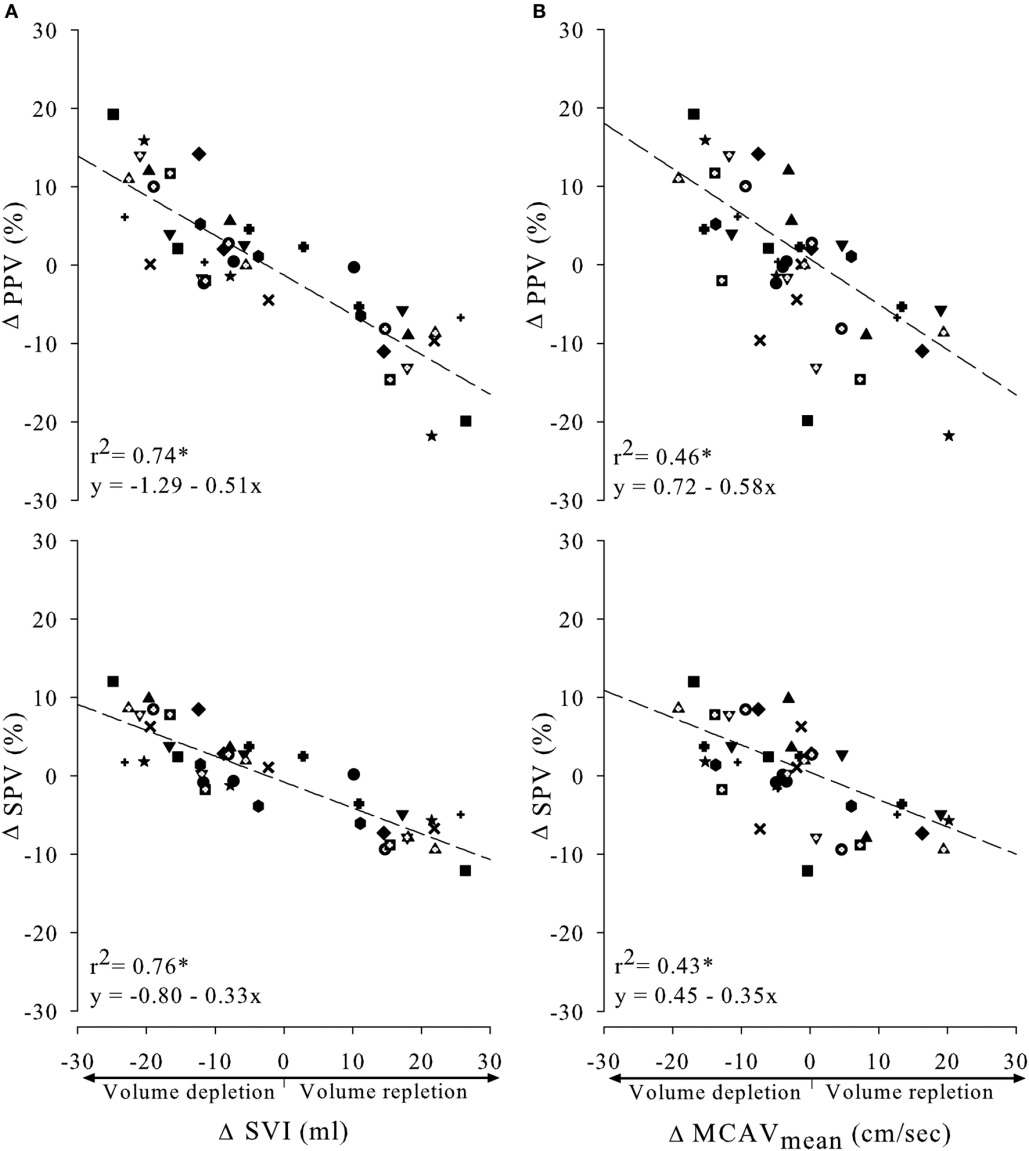
**Relationship between absolute change in pulse or systolic pressure variation (PPV and SPV, respectively), and absolute change in SVI (A) or MCAV_mean_ (B)**. All subjects were indicated by symbols and contributed three points in each regression line (during 30 and 70 degrees HUT + 0 degrees during tilt back). ^*^p < 0.0001.

Figure 6, panel (A) displays single linear regression plots between ΔSVI and ΔPPV or ΔSPV. Regression analysis between ΔSVI and ΔPPV/ ΔSPV showed linear correlations (PPV: *r*^2^ = 0.74, *p* < 0.0001 and SPV: *r*^2^ = 0.76, *p* < 0.0001) with a higher slope for ΔPPV compared to ΔSPV. Single linear regression analysis was also applied on ΔMCAV_mean_ and ΔPPV or ΔSPV [see panel (B) of Figure 6]. Correlations were seen between ΔPPV/ ΔSPV and ΔMCAV_mean_ (PPV: *r*^2^ = 0.46, *p* < 0.0001 and SPV: *r*^2^ = 0.43, *p* < 0.0001). The slope of the regression plots for both ΔPPV and ΔSPV were comparable with those between ΔPPV/ ΔSPV and ΔSVI. Again, the highest slope was seen for ΔPPV.

**Figure 7:**
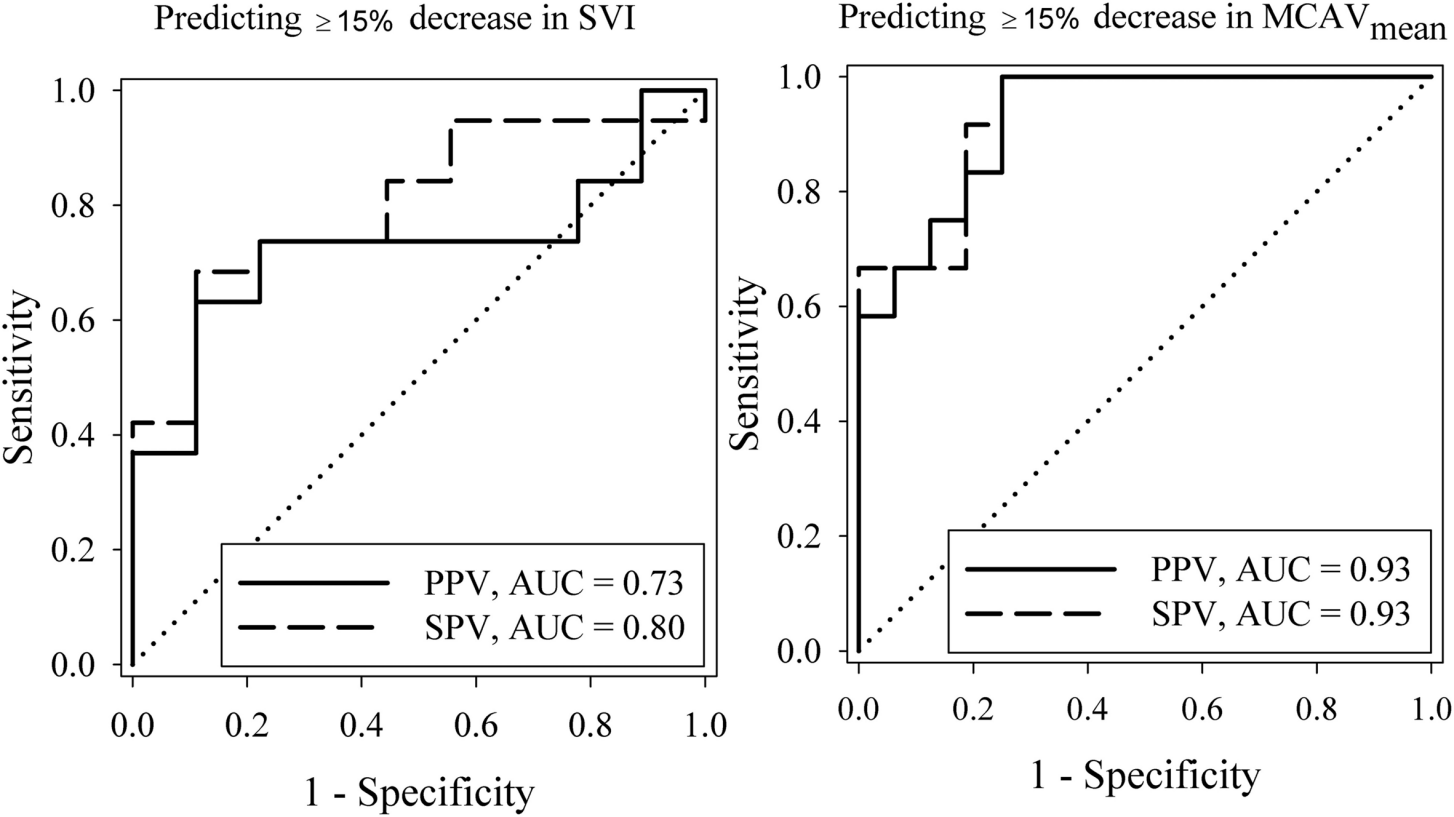
**Receiver operating characteristics (ROC) curves for the performance of PPV and SPV in predicting ≥15% decrease in SVI (left) and ≥15% decrease in MCAV_mean_ (right)**. PPV, pulse pressure variation; SPV, systolic pressure variation; AUC, area under curve; SVI, stroke volume index; MCAV_mean_, mean middle cerebral artery velocity.

**Figure 8:**
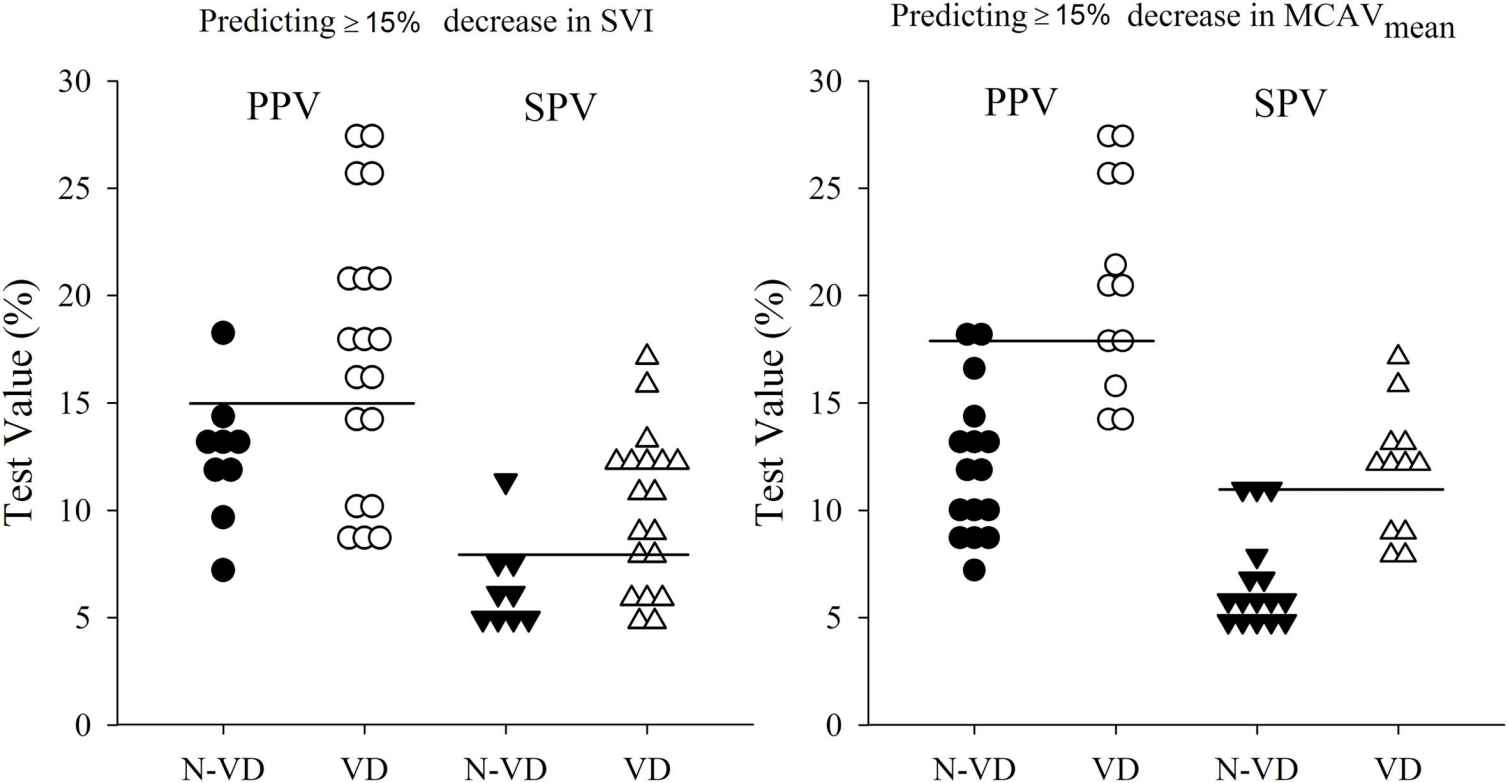
**Dot histograms of having a volume deficit (VD) or not having a volume deficit (N-VD) defined by ≥15% HUT induced decrease in SVI (left) or MCAV_mean_ (right)**. PPV is depicted by the circles and SPV is depicted by the triangles. The cutoff threshold values are represented as a horizontal line and described in Table 2. PPV, pulse pressure variation; SPV, systolic pressure variation; SVI, stroke volume index; MCAV_mean_, mean middle cerebral artery flow velocity.

In Figure 7, ROC curves for the performance of arterial pressure variations in predicting ≥15% decrease in SVI (left panel) and ≥15% decrease in MCAV_mean_ (right panel) are shown. The AUC values were the highest for PPV and SPV when predicting a decrease in MCAV_mean_ compared to predicting a decrease in SVI (PPV: 0.93 vs. 0.73 and SPV: 0.93 vs. 0.80). The cutoff thresholds and its corresponding sensitivity and specificity values are described in Table 2. PPV and SPV predicted a ≥15% reduction in MCAV_mean_ and SVI with comparable sensitivity (67/67% vs. 63/68%, respectively) and specificity (94 vs. 89% for both PPV and SPV). In Figure 8, dot histograms associated with the ROC curves are shown for the prediction of ≥15% decrease in both SVI and MCAV_mean_.

**Table 2:** **Predictive value of PPV and SPV for (changes in) SVI and MCAV_mean_ with cutoff thresholds and corresponding sensitivity of specificity**.

		**Cutoff threshold**	**Sensitivity (%)**	**Specificity (%)**
≥15% change in SVI	PPV	>15	63	89
	SPV	>8	68	89
≥15% change in MCAV_mean_	PPV	>18	67	94
	SPV	>11	67	94

PPV, pulse pressure variation; SPV, systolic pressure variation; SVI, stroke volume index; MCAV_mean_, mean middle cerebral artery flow velocity.

### Dynamic CA

**Figure 9:**
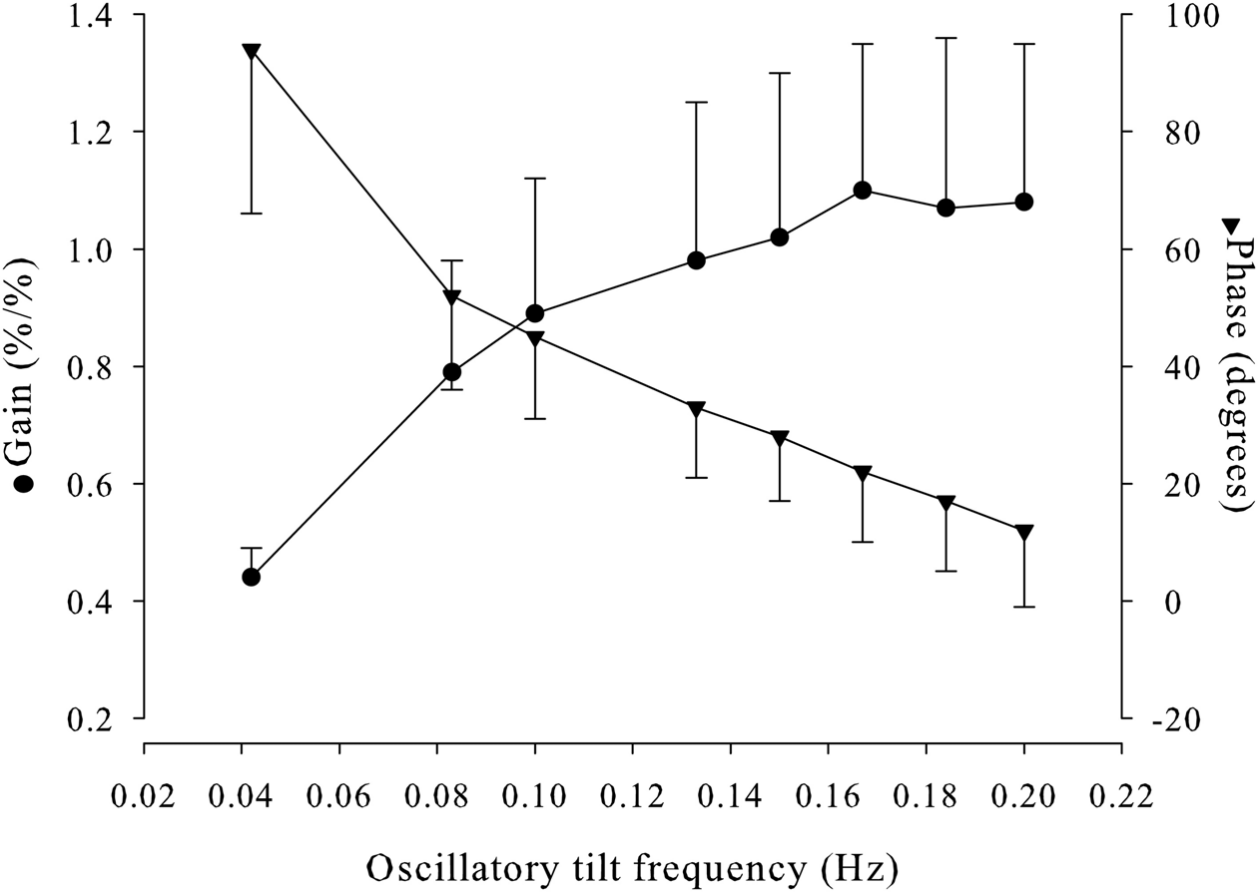
**MCAV_mean_-MAP transfer function displayed by gain and phase for each oscillatory tilt frequency**. MCAV_mean_, mean middle cerebral artery flow velocity; MAP, mean arterial pressure.

The MCAV_mean_-to-MAP at brain level transfer functions displayed the expected high-pass filter characteristics of dynamic autoregulation, with a decrease in phase lead and increase in normalized gain with increasing sinusoidal tilt frequency (Figure 9). Phase and gain at 0.1 Hz were, respectively, 45 degrees and 0.89%/%.

## Discussion

The main new finding of this study is that in spontaneously breathing subjects under conditions of depletion and repletion of CBV, MCAV_mean_ was linearly related to arterial pressure variations. Specifically, arterial pressure variations predicted a decline in MCAV_mean_ and SVI with comparable sensitivity and specificity.

### Posture and central blood volume depletion

Clinically, hypovolemia is manifested by a reduced CBV. With passive HUT, approximately 700 ml of CBV redistributes from the chest into the gravitational dependent regions, largely contained in the venous compartment and therefore not contributing effectively to the circulating blood volume (Sjöstrand, 1953; Rowell, 1986). In this study, a postural reduction in CBV coincided with a decline in SVI (Friedman et al., 1990; Matzen et al., 1991; Pawelczyk et al., 1994; Cai et al., 2000). This is attributed to blood pooling in the lower parts of the body and to a reduction in venous return which is in agreement with data from earlier studies (Harms et al., 2003; Immink et al., 2009). Constant values of MAP by a baroreflex mediated increase in TPR counterbalancing the postural reduction in SVI and CI illustrates the contention that MAP does not reflect changes in CBV (Van Lieshout and Wesseling, 2001; Secher and Van Lieshout, 2005).

### Posture and PCO_2_

Increased ventilation and corresponding lowering of PaCO_2_ associated with postural stress, is considered to be contributory to the reduction in MCAV_mean_ (Kapoor, 2002; Chen-Scarabelli and Scarabelli, 2004; Donnelly et al., 2011). The mechanisms that drive breathing during postural stress are not well understood but likely find their origin in both the brain and the periphery. We earlier considered that in the upright position the larger BP variability and less stable blood flow enhance fluctuation of PaCO_2_ as an input signal to the carotid body chemoreceptors (Immink et al., 2013). The interaction of enhanced baroreceptor activity and carotid body chemoreceptor stimulation may modify the respiratory drive (Biscoe and Purves, 1967a,b). Arterial hypocapnia has been associated with orthostatic intolerance and lowering of PaCO_2_ may reduce the prevailing peripheral vasomotor tone (Shoemaker et al., 2001). Thus, the postural reduction in PetCO_2_ suggests a contribution of mild hypocapnia to the reduction in cerebral perfusion. We consider that PetCO_2_ tracks changes in arterial carbon dioxide tension (PaCO_2_) in a fixed body position only, whereas the PaCO_2_ -to- PetCO_2_ gradient is enhanced by the postural reduction in CO. This results in an increased VE/Q ratio (Riley et al., 1959; Gisolf et al., 2004b; Immink et al., 2006) with overestimation of the reduction in P_a_CO_2_ (Immink et al., 2006, 2009). Also, when during passive head-up tilt PetCO_2_ is clamped to the level in the supine position, MCA *V*_mean_ declines in the first minute of tilt only. Afterwards the postural reduction in MCA *V*_mean_ has become independent of the ~4 mmHg reduction in PetCO_2_ for at least 5 min in the HUT 70 position (Immink et al., 2009). In the present study, postural stress, duration of tilt and reduction in PetCO_2_ were comparable to the unclamped limb in that study supporting that the decrease in PetCO_2_ during HUT does not explain the reduction seen in MCAV_mean_.

### Posture and cerebrovascular autoregulation

The decline in CPP is explained by the HUT induced hydrostatic pressure gradient when the cerebral circulation is positioned above the level of the heart. According to the traditional concept of CA, cerebral blood flow (CBF) is maintained more or less constant in the face of changing CPP (Roy and Sherrington, 1890; Lassen, 1974). Nevertheless, postural stress elicits reductions in indices of CBF irrespective of the fact that CPP remains within the so called autoregulatory range, challenging the concept of CA as a plateau (Immink et al., 2010; Lucas et al., 2010). In fact, constant CBF would require an infinite gain which is generally not operative in humans (Van Lieshout et al., 2003; Panerai, 2004; Immink et al., 2013; Willie et al., 2014). Assessment of CA is based on introducing CCP fluctuations and quantifying their transfer to the blood velocity in a large cerebral artery in terms of phase angle and gain. This approach addresses specifically the dynamic component of CA. CA is, however, considered to encompass both static (long-term) and dynamic (short-term) components (Van Lieshout et al., 2003). It remains unclear whether short- and long-term regulation of CBF are separate mechanistic entities (Ainslie and Brassard, 2014). Reference values have not been defined and overlap exists between healthy subjects vs. patients with impaired dynamic CA. We demonstrated the expected high-pass filter characteristics and therefore assume that in the young adult subjects in this study dCA was intact. However, CA integrity does in itself not preclude some influence of the postural reduction in CPP on MCAV_mean_. Of interest, the postural reduction in CPP was only minor and the correlation between changes in CPP and MCAV_mean_ weak, supporting that the effect of the reduction in CPP on MCA flow velocity, if anything, must have been limited.

### Cerebral blood flow velocity and stroke volume

Cerebral blood flow velocity and its relation with CO has been investigated in several studies. Under conditions of 30 Torr lower body negative pressure (Zhang et al., 1998) and HUT (Jorgensen et al., 1993) both CO and MCAV_mean_ decreased whereas in response to moderate exercise (Brys et al., 2003; Ogoh et al., 2005) CO and MCAV_mean_ increase together with CBV without changes in PaCO_2_. In this study, the change in CI from 0 to 30 degrees HUT was limited. From 30 to 70 degrees HUT, SVI and CBV decreased further but CO was maintained, probably attributable to the baroreflex mediated increase in HR. During progressive central hypovolemia a reduction in MCAV_mean_ coincided with the fall in SVI, and vice versa MCAV_mean_ and SVI both increased in response to a simulated fluid challenge by repositioning from upright to supine, supporting a dependency of MCAV_mean_ on CBV. Furthermore, SVI appeared to be the strongest hemodynamic predictor for changes in MCAV_mean_. Collectively, these findings suggest that in spontaneously breathing volunteers subjected to simulated progressive central hypovolemia, changes in SVI reflect those in MCAV_mean_ in a linear manner (see Figure 4).

### Predictive value of arterial pressure variations

During surgery or in an intensive care setting, an increase in SVI in response to fluid administration in the anesthetized patient is considered to indicate fluid responsiveness. This study demonstrated an increase in arterial pressure variations in response to a clinical relevant decrease in SVI during HUT and vice versa during tilt back. This strong correlation between SVI and arterial pressure variations is in agreement with earlier research (Hofer et al., 2005; Jacques et al., 2011). A new finding is that under the conditions of this study MCAV_mean_ and arterial pressure variations are related too.

Although arterial pressure variations have been proven valuable to predict fluid responsiveness in patients receiving mechanical ventilation, their predictive value in spontaneously breathing patients is lower (Bendjelid and Romand, 2003; Heenen et al., 2006; Soubrier et al., 2007). Our findings are in agreement with these data, whereas the present study extends this knowledge by demonstrating that arterial pressure variations predict changes in MCAVmean with comparable sensitivity and specificity values, and a higher accuracy during graded hypovolemia. Extrapolating this to clinical practice, a fluid challenge targeting SVI also targets brain perfusion.

Potential limitations inherent to the study design should be considered. Transcranial Doppler ultrasonography is used to monitor changes in CBF. This technique has been widely used under the assumption that the cross-sectional area of the MCA is maintained during the measurement. Possible changes in the diameter of the insonated vessel by enhanced sympathetic activity could modulate velocity independently of flow. Previous research showed that increases in sympathetic outflow by baroreflex disengagement or chemoreflex activation do not alter MCA diameter (Serrador et al., 2000), and we therefore assume that a constant MCA diameter links changes in cerebral blood velocity to changes in flow.

Detecting a volume deficit is considered as the major goal of determining fluid responsiveness. However, recent studies indicate that under certain conditions fluid bolus administration is associated with an increased mortality (Maitland et al., 2011). It is recognized that fluid administration should be practiced with much greater caution and increased vigilance and a more conservative fluid management seems appropriate (Myburgh, 2011). The values represented in Table 2 are therefore optimized for high specificity rather than high sensitivity.

In summary, the present study shows for the first time in awake humans subjected to progressive central hypovolemia that arterial pressure variations are related to both CBF velocity and SVI. Specifically, PPV and SPV predicted changes in both SVI and MCAV_mean_ with comparable sensitivity and specificity, however the predictive value is limited in spontaneously breathing subjects.

## Author contributions

Anne-Sophie G. T. Bronzwaer contributed to the experimental design, data acquisition, data analysis and writing the manuscript. Wim J. Stok contributed to data acquisition, data analysis and manuscript revision. Berend E. Westerhof contributed to the experimental design, data analysis and manuscript revision. Johannes J. van Lieshout supervised the study, contributing to the experimental design, data analysis and writing the manuscript.

### Conflict of interest statement

The authors declare that the research was conducted in the absence of any commercial or financial relationships that could be construed as a potential conflict of interest.
